# Reliable Attention Network Scores and Mutually Inhibited Inter-network Relationships Revealed by Mixed Design and Non-orthogonal Method

**DOI:** 10.1038/srep10251

**Published:** 2015-05-21

**Authors:** Yi-Feng Wang, Xiu-Juan Jing, Feng Liu, Mei-Ling Li, Zhi-Liang Long, Jin H. Yan, Hua-Fu Chen

**Affiliations:** 1 Key laboratory for NeuroInformation of Ministry of Education, School of Life Science and Technology and Center for Information in BioMedicine, University of Electronic Science and Technology of China, Chengdu, 610054, China; 2 Tianfu College, Southwestern University of Finance and Economics, Chengdu, 610052, China; 3 Department of Radiology and Tianjin Key Laboratory of Functional Imaging, Tianjin Medical University General Hospital, Tianjin, 300052, China; 4 Center for Brain Disorders and Cognitive Neuroscience, School of Medicine, Shenzhen University, Shenzhen, 518060, China

## Abstract

The attention system can be divided into alerting, orienting, and executive control networks. The efficiency and independence of attention networks have been widely tested with the attention network test (ANT) and its revised versions. However, many studies have failed to find effects of attention network scores (ANSs) and inter-network relationships (INRs). Moreover, the low reliability of ANSs can not meet the demands of theoretical and empirical investigations. Two methodological factors (the inter-trial influence in the event-related design and the inter-network interference in orthogonal contrast) may be responsible for the unreliability of ANT. In this study, we combined the mixed design and non-orthogonal method to explore ANSs and directional INRs. With a small number of trials, we obtained reliable and independent ANSs (split-half reliability of alerting: 0.684; orienting: 0.588; and executive control: 0.616), suggesting an individual and specific attention system. Furthermore, mutual inhibition was observed when two networks were operated simultaneously, indicating a differentiated but integrated attention system. Overall, the reliable and individual specific ANSs and mutually inhibited INRs provide novel insight into the understanding of the developmental, physiological and pathological mechanisms of attention networks, and can benefit future experimental and clinical investigations of attention using ANT.

Recent cognitive and neuropsychological research highlighted the heterogeneity of attention system^1^. In this framework, Posner and Petersen^2^ divided the attention system into three networks that carry distinct functional demands. The alerting (*A*) system initiates and sustains a readiness in reaction to upcoming stimuli; the orienting (*O*) component mediates the selection of relevant information for processing; and the executive control (*E*) network is involved in error monitoring and conflict resolving^3^,^4^. The efficiency and independence of attention networks were widely measured with the attention network test (ANT) which was devised by Fan and colleagues^5^.

Using the ANT and its revised versions^6^,^7^,^8^, the attention network scores (ANSs) and inter-network relationships (INRs) have been measured and applied in a wide range of investigations such as development, psychiatric disorders, neuroimaging, and genetics^3^,^4^,^9^. However, the low reliability (split-half reliability: 0.14–0.71; test-retest reliability: 0.35–0.80) of ANSs may decrease statistical power, underestimate the strength of true inter-network correlation, and restrict the application of ANT in developmental and clinical studies^10^,^11^. In addition, the ANSs and INRs are undetected in a large proportion of studies^10^,^12^. Considering the widely usage of ANT in recent investigations about developmental differences, individual differences, and deficits in special populations^3^,^13^, improving the reliability of ANSs and illuminating the pattern of INRs are important and urgent tasks.

There are at least two methodological reasons that might be responsible for the instability of ANT. First, in the event-related design, the inter-network interplay would influence the efficiency of each network, because the present attention operation strongly depends on near past experience^13^,^14^. Allocating different networks into separate blocks/runs may avoid inter-network impact from past trials^15^. Thus, we revised the ANT with a mixed block/event-related design. In the mixed design, ANSs and INRs were tested in separate runs to avoid the influence from other components as much as possible when measuring one network or interaction. Second, orthogonal contrasts used in the ANT are susceptible to INRs^10^. Although the non-orthogonal method has been used to calculate ANSs^12^,^13^, these results were still affected by INRs in the event-related design. We suggested that the combination of non-orthogonal method and mixed design is more reasonable to accurately measure the ANSs.

With the mixed design, we allocated the measurement of ANSs and interactions into six separated blocks: three effect blocks were used to test three ANSs, respectively; three interaction blocks were used to examine pair-wise INRs. Specifically, spatial targets were excluded when measuring *A* and *E* to avoid the influence from *O*; cues were canceled when testing *O* and *E* to exclude the alerting effect; incongruent targets were eliminated when examining *A* and *O* to avoid the conflict effect. Two significant modifications should be noted. First, all effects measured here were stimulus-driven. The exogenous attention and endogenous attention have different time courses and neural mechanisms^16^,^17^. They are involved in previous measurements of ANSs and INRs to varying degrees, bringing difficulties in explaining ANSs and INRs. We endeavored to avoid the confounding of exogenous and endogenous mechanisms from informative spatial cue^18^ and varying cue-target intervals^17^ by eliminating spatial cue and using a constant cue-target interval of 300 ms^16^. Second, the effects of *A* and *O* were separated by removing cues when testing *O* rather than using a cross-modality operation^7^, because attention has been demonstrated to be modality dependent^19^,^20^. Furthermore, in the interaction version of ANT, both alerting tone and spatial cue may enhance phasic alerting^12^. To avoid inter-network interference and get more accurate measurements of ANSs and INRs, we allocated different ANSs and INRs into separate blocks/runs with a mixed design.

The non-orthogonal method was adopted to further avoid inter-network interference. Because of the interplay among diverse cue and target conditions, ANSs obtained by orthogonal and non-orthogonal methods were significantly different^12^,^13^. These researchers, therefore, suggested that the non-orthogonal method is better than the orthogonal method to get single measurements of ANSs. In the current study, nine equations were utilized to calculate ANSs and directional INRs. Combined with mixed design, this non-orthogonal method would reduce within-subject variability by avoiding inter-network interferences, thus obtaining more reliable and unsullied ANSs and directional INRs.

By separating the measurements of ANSs and inter-network interactions, we aimed to obtain more reliable and accurate ANSs and INRs. This endeavor would benefit investigations about the psychological and physiological mechanisms of attention and the application of ANT.

## Results

Table 1 shows the RT and accuracy of each condition. Because the ceiling effect was evident, values of accuracy were not used for further analysis. Since RTs were not normally distributed, the median RT per condition was used for computing ANSs and directional INRs^21^. We replaced RTs with the median for trials with error responded (0.97%) or with the RT larger than 3 standard deviations (1.17%) in each cue-target condition in each subject. We replaced these data due to two reasons: 1) there are only 24 pairs of trial for each ANS which is much fewer than previous studies (e.g., 72 or 96 pairs of trial in the original version of ANT); 2) the analysis of split-half reliability requires equal numbers of trial for two halves.

### ANSs and directional INRs

**Figure 1:**
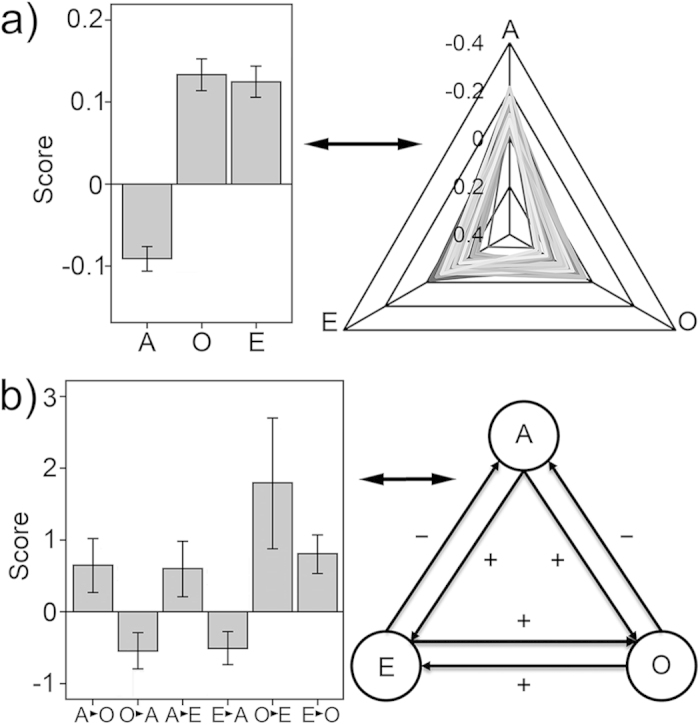
The attention network scores (ANSs) and directional inter-network relationships (INRs). (**A**) The mean ANSs (left) and their profiles on each subject (right). The *A* benefited RT, whereas *O* and *E* prolonged RT. Each person has a specific profile of *A-O-E*. (**B**) The mean INRs (left) and a simple framework of INRs (right). Both *O* and *E* reduced the effect of *A*. *A* enhanced effects of *O* and *E*. The effects of *O* and *E* were mutually promoted. *X→Y*: the influence from *X* to *Y.* Positive value represented the increase in RT, while negative value expressed the decrease in RT. Error bar shows the 95% confidence interval.

As shown in Table 2 and Fig. 1, all ANSs and directional INRs were remarkably different from zero. For ANSs, obvious decrease in RTs could be seen on central cue condition than on no cue condition; while RTs were significantly increased on spatial target and incongruent target conditions than on central target and congruent target conditions, respectively. As shown in Fig. 1B, the *O* and *E* impaired the efficiency of *A*, indicating a weakened alert on spatial target and incongruent target conditions. The *A* enhanced the efficiencies of *O* and *E*, indicating slowed orienting and weakened executive control capacity in cued trials. The effects of *O* and *E* mutually enhanced, indicating slowed orienting on the incongruent target condition and impaired executive control capacity on the spatial target condition. These directional INRs revealed remarkable inter-network inhibition in interaction blocks, suggesting that the non-orthogonal method and mixed design were necessary to avoid inter-trial and inter-network interferences in measuring ANSs.

### Inter-network interactions

**Figure 2:**
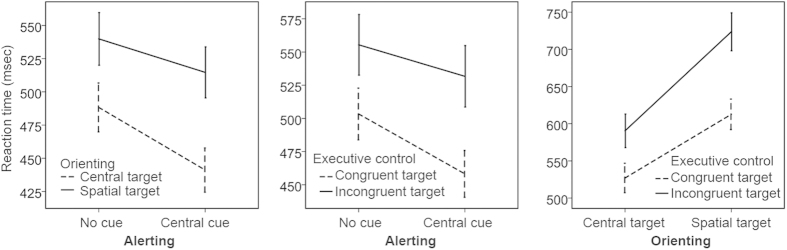
Inter-network interactions. Pairwise interactions were measured in separate blocks. All interactions of alerting × orienting, alerting × executive control, and orienting × executive control were significant. Specific inter-network relationships were shown in Fig. 1. Error bar shows the 95% confidence interval.

As shown in Table 3, both main effects and interactions for three pairs of network were extremely significant, indicating strong coordination between networks when the cognitive system operates more than one network. Visualized inter-network interactions could be seen in Fig. 2. Post hoc analysis was not performed because the directional relationship analysis had described INRs in detail.

### Inter-network correlations

For ANSs, Pearson correlation analysis showed a weak negative correlation between *A* and *O*: *r* = –0.310, *P* = 0.040. However, this correlation could not pass Bonferroni adjustment^22^. The correlations between *A* and *E* (*r* = 0.174, *P* = 0.257) and between *O* and *E* (*r* = 0.045, *P* = 0.772) were far from significance. These results revealed that the three networks are mutually independent.

**Figure 3:**
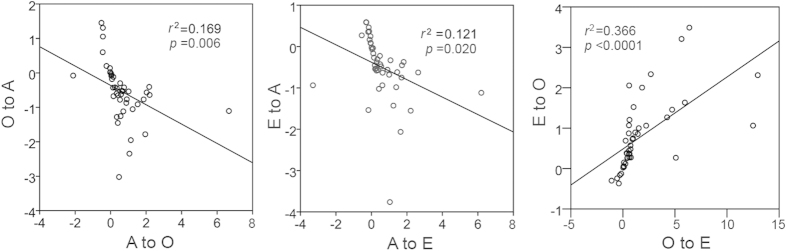
The correlations between directional inter-network relationships. If one network exerts more influence on another one, the reverse impact is increased simultaneously.

For directional INRs, striking negative correlations between *A→O* and *O→A* (*r* = –0.411, *P* = 0.006) and between *A→E* and *E→A* (*r* =–0.348, *P* = 0.020), and positive correlation between *O→E* and *E→O* (*r* = 0.605, *P* < 0.0001) were observed (Fig. 3). Of note, the negative correlation was due to the negative value of *A*. These significant correlations indicated that the networks were mutually inhibited when two networks were operated simultaneously.

### Reliability

The split-half reliabilities of *A* (0.684), *O* (0.588), and *E* (0.616) were all over 0.5. Considering that the split-half reliabilities of *A* (0.14–0.27) and *O* (0.26–0.38) were much lower than 0.5 in most studies^10^, the high reliabilities of three networks in the current study, therefore, have positive theoretical and clinical values.

## Discussion

The reliability of ANSs and independency of networks suffered a long-term debate since Posner and Peterson^2^ put forward the three networks of attention system. Combined the non-orthogonal method with mixed design, we observed significant ANSs and INRs, indicating a differentiated but integrated attention system. Although with a few trials, we got very high split-half reliabilities of ANSs, suggesting that our method has a strong potential in measuring ANSs in developmental and clinical investigations.

The ANSs are significant and mutually independent. The lack of correlations among ANSs indicates that each person has a specific profile of *A-O-E* (Fig. 1A, right panel). In other words, a person with high *O* efficiency does not necessarily have high *E* efficiency, indicating separate mechanisms for three networks. Previous studies have demonstrated that the differentiation of three attention networks takes place in early ages. Specifically, the *O* matures at about 4 years^1^; the *E* gradually differentiates from *O* and matures during puberty^23^; the *A* gradually develops and matures during adolescence^24^. Essentially, the three networks are driven by different genes^9^ and neurotransmitters^3^,^25^. These genetic, developmental, and physiological factors may cooperate to create a specific profile of *A-O-E* on each person.

The independence and between subjects variability of ANSs are also supported by selective deficit in a variety of diseases. For example, specific *E* deficits have been observed in individuals with borderline personality disorder^26^ and posttraumatic stress disorder^27^. A specific *O* deficit has been reported in individuals with concussion^28^. A specific *A* deficit^29^ or both *O* and *E* deficits^30^ have been reported in different schizophrenia groups. The selective impairment of one or more networks indicates that the profile of *A-O-E* could serve as a psychological marker of a wide range of diseases^31^. However, these reports of selective impairments rely on the independence and reliability of ANSs^10^. The high reliability and remarkably independent ANSs in the present study suggested that combining non-orthogonal contrast with mixed design can provide a better measurement of the profile of *A-O-E* than previous studies and thus can benefit future theoretical and clinical studies.

The reliability of ANSs is particularly important given the widely application of ANT in developmental and clinical studies. Low reliability of *A* and *O* in previous studies not only fails to meet the demand of clinical investigations but also decreases statistical power and underestimates true inter-network correlations^10^,^11^. We suggest that the reliability of ANSs is constrained by at least three factors except for methodological factors announced in the Introduction section. First, the infra-slow fluctuations of RT are associated with significant intra-individual variation^32^,^33^,^34^, resulting in low reliability^11^. Second, more trials may increase reliability^11^ but induce fatigue effect that prolongs and increases variability of RTs^35^. Third, the fluctuations of arousal may increase the variability of observed ANSs on the one hand, because the ANSs are obtained by contrasting different effects with the baseline state; and the true ANSs on the other hand, because the arousal interacts and endogenously contributes to all three networks^36^. Therefore, reducing inter-trial variability or bringing it into statistical analysis is necessary to improve the reliability of ANSs.

The interaction between *A* and *O* has been reported by some researchers^7^,^37^ but not by others^5^,^38^. Those studies reporting a speeded-up orienting of attention under conditions of high alerting have proposed that the influence from *A* to *O* occurs only within a short (<500 ms) time course and peaks at about 150 ms^7^,^17^. They hypothesized that the *A* benefits the *O*^7, 37^, but provided no evidence and assumption on the influence from *O* to *A*. So far, there is not a theoretical framework about the interplay between *A* and *O*.

Beyond the unidirectional influence from *A* to *O*, we observed a larger effect of *O* when there was an alerting cue and a smaller effect of *A* when spatial target was presented. With a close inspection, we found that the alerting cue improved the respond to central target more than to spatial target, whereas the spatial target increased RTs to cued trials more than to un-cued trials. Therefore, the influence from *A* to *O* in the present study is opposite to previous findings. This discrepancy can be coordinated by a cue-target consistency effect. The consistent cue-target condition improves not only the phasic alerting but also the target processing at the cued location^39^,^40^, supporting the interaction between *A* and *O*. For the influence from *A* to *O*, when a spatial cue is presented as in previous studies, the processing of the target at that location is improved than at other locations. Reversely, if an alerting signal cues a central position, the processing of the target at this location is improved. For the influence from O to A, if the target appears at the same location as the cue, the processing is improved; if it appears at a different location from the cue, the processing is impaired. It’s worth noting that, in previous studies, the cue validity and cue-target interval may also contribute to the interaction between *A* and *O* by modulating both *A* and *O* via expectancy and time variability^41^. As a promising theoretical framework about the relationship between *A* and *O*, the cue-target consistency effect should be verified by manipulating cue validity and cue-target interval in future studies with behavioral and neuroimaging techniques^25^.

There are several opinions on the interaction between alerting and executive control networks. First, Callejas *et al.*^7^ proposed that alertness directly inhibits executive control by impeding the function of anterior cingulate cortex. Second, Böckler *et al.*^42^ and Fischer *et al.*^43^,^44^ suggested that alerting cues amplify automatic response activation related to both the relevant and irrelevant task dimensions, thus enhancing the conflict effect. Third, McConnell and Shore^13^ suggested that alerting cues encourage diffusing attention between two possible target locations. The uncertainty of target location in the ANT disturbs the attention in a focused state, making it harder to ignore distracting stimuli. Fourth, Fan *et al.*^5^ assumed that the alerting condition reduced RTs, thus providing less time to process conflict. Fifth, Weinbach and Henik^45^,^46^ demonstrated that alerting cues expand the attention scope and create a bias to global processing, thus increasing the disturbance of distracting stimuli. These views highlighted the influence from *A* to *E*, whereas the resource competition explanation focused on the bidirectional relationship between *A* and *E*^6^. Accordingly, the *A* and *E* may compete resources from their shared brain regions, e.g., the anterior cingulate cortex and fronto-parietal network.

By eliminating spatial targets, we observed enlarged conflict effect in the alerting condition as well as reduced alerting effect in the incongruent condition. These results are consistent with the resource competition explanation^6^, and can be partially explained by other perspectives except for McConnell and Shore’s^13^ target uncertainty hypothesis. It’s worth noting that the resource competition hypothesis can also explain the mutual inhibition between *A* and *O*, and between *O* and *E*. Given the complex and flexible interaction between *A* and *E*, more factors should be considered in future investigations such as the spatial pattern of targets^46^, dopamine level^47^, task difficulty^36^ and arousal^48^. We suggest that investigations on bidirectional relationship between *A* and *E* are essential to illuminate the mechanisms of alerting and executive control systems.

There are different assumptions about the influence from *O* to *E* in previous studies^5^,^7^,^13^. Fan *et al.*^5^ explained it from the perspective of attention resource that prior orienting of attention reduced RTs, resulting in insufficient time to process conflict. Callejas *et al.*^7^ suggested that the prior orienting of attention to the target location could help subjects to concentrate on that area and ignore the incongruent flankers. McConnell and Shore^13^ assumed that orienting to another location induced the global processing strategy, leading to a difficult in ignoring flankers. The confliction among these hypotheses lies in how subjects use attention resource and focusing strategy when orienting to a new position.

In this study, RTs of *O* were longer in incongruent condition than in congruent condition, while conflict effect was larger in spatial target condition compared with the central target condition. When *O* and *E* occur simultaneously, the resource competition hypothesis predicts bidirectional impairment between them, while the focusing strategy hypothesis anticipates an inhibition from *O* to *E* and a promotion from *E* to *O* because both *E* and *O* induce a global processing strategy. Therefore, the current results are consistent with the resource competition perspective rather than the focusing strategy hypothesis.

Convergent evidence showed that the attention system is a differentiated but integrated system. First, there is no consensus on the correlations among ANSs^5^,^10^,^13^,^49^, indicating a large inter-individual variability of the *A-O-E* profile. With high reliability of ANSs, the inter-network correlation is more properly estimated in the current study than in previous studies with low reliability of ANSs^10^,^11^. The lack of inter-network correlation we observed in a high homogeneous sample (college students), therefore, strongly propose a large inter-individual variability of the *A-O-E* profile. Many factors such as gene, development, physiology, and the state during test may be responsible for the particular *A-O-E* profile of each participant^9^,^23^,^36^,^41^,^47^.

Second, as an integrated system, attention resources would be allocated flexibly among networks. The resource competition hypothesis can explain the current findings as well as results in previous studies^5^,^6^. Meanwhile, inter-network cooperation is essential to produce a more adaptive behavior^7^. This requires the attention system to balance the roles of three networks dynamically^37^,^41^ and use various strategies such as global/local processing^13^,^45^,^46^ and multisensory integration^7^,^40^,^49^.

The differentiated networks and inter-network integration of attention are supported by different neurotransmitters and partially overlapped brain regions^3^,^4^,^17^. We also show that the networks are independent when they are separately measured, but mutually impaired when they are operated simultaneously. The differentiation and bidirectional impact of attention networks are of importance for our understanding of various *A-O-E* profiles in developmental and clinical investigations. For instance, if the *E* is differentiated from *O*^23^, can we expect stronger influence from *O* to *E* than from *E* to *O* in children before three years old compared with elder children and juveniles? In addition, if individuals with borderline personality disorder have a specific *E* deficit^26^, can we anticipate alterations of directional influence between *E* and *A* and between *E* and *O*? Our method may provide an effective solution to these developmental and clinical mysteries. Furthermore, reliable measurements of ANSs and directional INRs in a simple context are vitally important in developmental and clinical studies.

Combining non-orthogonal method with mixed design, we obtained independent ANSs with high reliability and remarkable bidirectional INRs. However, there are several limitations. First, the attention system is too complex to be measured by a single task. For example, several dichotomies have been highlighted in recent studies such as exogenous/endogenous attention^17^, tonic/phasic alerting^46^, top-down/bottom-up control^14^, and spatial/objective (non-spatial) attention^46^. Although the ANT tests a basic model of attention, complex relationships among more subcomponents should be examined in detail with reasonable reliability. Second, the cue-target interval in the present study is constant, eliminating the interference from temporal expectancy^25^,^41^. However, the inter-network relationship has been demonstrated to be time and probability dependent^37^,^41^. Cue validity, inter- and intra-trial possibilities, and more cue-target intervals are warranted in future studies to explore complex INRs. Third, our subjects were all college students with high homogeneity. This may not well reflect between-subject variability and the development and aging of attention system. A large sample size involves children, adults and old people may help us to dissociate between- and within-subject variability. Last, variables (e.g., sleep hours, time of testing, and drug consumption) who influence attention state should be recorded in future studies.

## Conclusions

Compared with previous studies, we used fewer trials to produce higher reliability of ANSs. Meanwhile, we demonstrated a differentiated but integrated attention system. Specifically, the three networks are independent but mutually inhibited when two networks are operated simultaneously. The mutual inhibition was in line with the resource competition hypothesis. The high reliability of ANSs and directional INRs may shed light on the developmental, degenerative, and pathological mechanisms of attention networks and benefit the application of ANT in developmental and clinical studies.

## Material and methods

### Subjects

Fifty subjects took part in the experiment at extracurricular time (29/21 males/females, reported right-handed, ages ranged from 18–29 years with mean age of 20.34 ± 2.86). All participants had regular routines and were asked to have enough sleep and avoid from any drugs and alcohol in 12 hours before the experiment. All participants reported normal or correct-to-normal vision, without any medication, and neurological or psychiatric disorders. Six subjects were excluded from subsequent analyses due to following reasons: one for misunderstanding the introduction, two for procedure erroneous, and three for low accuracy (<80%) in any condition.

### Ethics Statement

This study was approved by the research ethical committee of School of Life Science and Technology, University of Electronic Science and Technology of China. The methods were carried out complied with the tenets of the Declaration of Helsinki. All the participants gave their informed consents prior to their inclusion in the study.

### Apparatus and Stimuli

The experiment was run on a Lenovo-PC computer with a 20-inch color screen monitor. E-Prime 2.0 software (http://www.pstnet.com; Psychology Software Tools, Inc) was used for programming, display of stimuli, and timing control. Responses were collected through the computer keyboard. All stimuli were black figures presented at the center of a screen on a gray background. Fixation was a plus sign and subtended 0.22° visual angle. The length of the whole target (five arrows and inter-arrow blank) was 3.1°. The length of each arrow was 0.53° visual angle. The spatial target was 2° from the fixation.

### Procedure and design

**Figure 4:**
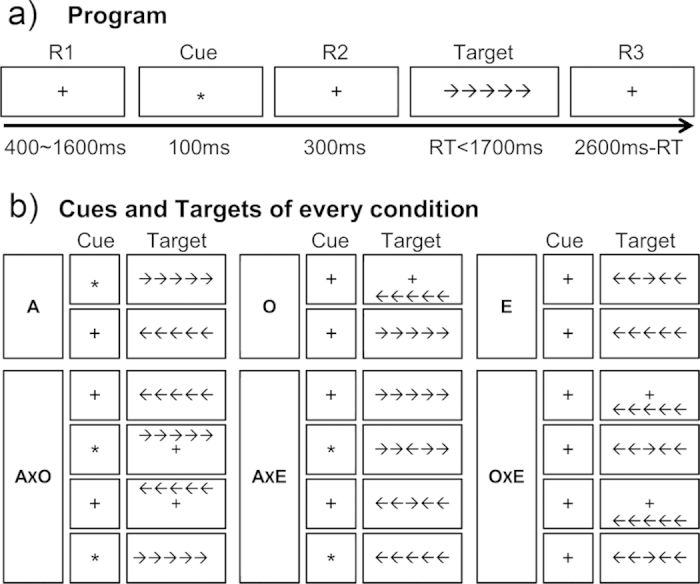
The program of the mixed design attention network test consisted of six blocks. The effects of alerting (*A*), orienting (*O*), and executive control (*E*) networks were tested in separated blocks to avoid interactions among them. The inter-network relationships were also assessed in independent blocks. For *A* and *E*, central targets were used to exclude the orienting effect; for *O* and *E*, cues were canceled to exclude the alerting effect; for *A* and *O*, incongruent targets were eliminated to avoid the conflict effect.

All participants completed six blocks of the mixed design attention network test. The order of six blocks was randomized. Each effect block contained 4 practice trials and 48 experimental trials, while each interaction block included 4 practice trials and 96 experimental trials. Eighteen cue-target conditions were involved in the task. This design advanced our previous study^12^ to all directional INRs. Participants were seated 70 cm from the monitor and were asked to focus on the fixation throughout the experiment and, during practice. As shown in Fig. 4, each trial began with a fixation lasting for 400–1000 ms. A 100 ms fixation (for *O*, *E* and half of the trials of *A*) or asterisk (for half of the trials of *A*) was followed. After a 300 ms delay, the target (central or spatial, congruent or incongruent) was presented for 1700 ms or lasted until the participant pressed a key. Lastly, another delay was presented to insure the overall time of the trial from cue to the end of the trial was 3000 ms.

Instead of the conventional subtraction measure^5^,^6^, we used ratio scores to define the efficiency of attention networks and INRs. The ratio scores could avoid the baseline difference and isolate the attention system from the overall reaction time (RT)^21^,^50^. The ANSs are listed in equations (1, 2, 3) using conditions in effect blocks.


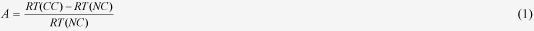



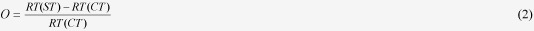



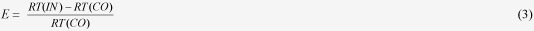


The INRs are calculated using conditions in interaction conditions with equations (4, 5, 6, 7, 8, 9). This method allows us to test the direction of impact beyond interaction, promoting our understanding of the relationship among attention networks.


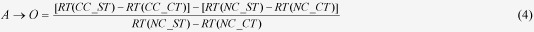



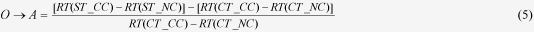



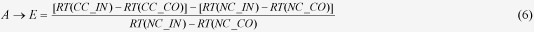



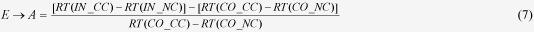



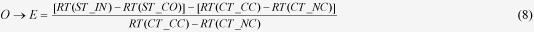



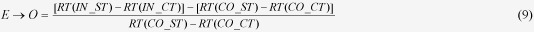


The abbreviations and the trials or effects they stand for are listed as follows: CC = central cue; NC = no cue; CT = central target; ST = spatial target; CO = congruent target; IN = incongruent target; *A* = alerting effect; *O* = orienting effect; *E* = executive control effect; *X→Y* =the influence from *X* to *Y*.

In these equations, the negative effect denoted beneficial in RT, whereas the positive effect represented cost in RT. This provided us an intuitive illumination of ANSs and INRs.

### Statistical analysis

All ANSs and INRs were computed based on RTs. The efficiency of ANSs and INRs was tested with one-sample *t*-test. The independency of three networks was examined using repeated measures analysis of variance (ANOVA). The relationships among ANSs and between INRs of each pair of network were assessed by Pearson correlation. Furthermore, the split-half reliability of three ANSs was estimated with a Monte Carlo simulation. Specifically, for each ANS, the 24 trials of each condition in each subject were randomly split into two halves 10000 times. The ANS was calculated in each of the two halves. The reliability was computed based on the 10000 pairs of ANS in forty-four subjects. The final reliability was the mean of 10000 correlation coefficients.

## Author Contributions

Y.F.W., X.J.J., J.H.Y. and H.F.C. designed the experiment; Y.F.W. and X.J.J. performed the experiment; Y.F.W., X.J.J., F.L., M.L.L. and Z.L.L. analyzed the data; Y.F.W., X.J.J., F.L., J.H.Y. and H.F.C. wrote the manuscript. All authors reviewed the manuscript.

## Additional Information

**How to cite this article**: Wang, Y.-F. *et al*. Reliable Attention Network Scores and Mutually Inhibited Inter-network Relationships Revealed by Mixed Design and Non-orthogonal Method. *Sci. Rep.* **5**, 10251; doi: 10.1038/srep10251 (2015).

## Figures and Tables

**Table 1:** The reaction time and accuracy per condition.

**Effect**	**Condition**	**Reaction time**	**Accuracy**
*A*	NC	479.063 (57.942)	0.998 (0.009)
	CC	434.750 (52.614)	0.992 (0.016)
*O*	CT	480.744 (58.559)	0.997 (0.011)
	ST	544.421 (69.706)	0.993 (0.020)
*E*	CO	489.909 (55.435)	0.996 (0.012)
	IN	550.142 (61.836)	0.991 (0.020)
*A* × *O*	NC_CT	488.267 (60.470)	0.996 (0.012)
	NC_ST	539.892 (65.214)	0.992 (0.021)
	CC_CT	441.097 (54.799)	0.992 (0.019)
	CC_ST	514.653 (63.084)	0.988 (0.025)
*A* × *E*	NC_CO	503.330 (64.014)	0.999 (0.006)
	NC_IN	555.432 (74.988)	0.988 (0.023)
	CC_CO	458.205 (57.942)	0.999 (0.006)
	CC_IN	531.699 (76.078)	0.975 (0.041)
*O* × *E*	CT_CO	527.011 (65.145)	0.998 (0.009)
	CT_IN	590.483 (73.913)	0.988 (0.025)
	ST_CO	612.632 (67.302)	0.994 (0.014)
	ST_IN	723.625 (83.658)	0.948 (0.058)

Note. Reaction time is in milliseconds. Standard deviations are in parenthesis. CC = central cue; NC = no cue; CT = central target; ST = spatial target; CO = congruent target; IN = incongruent target; *A*: alerting effect; *O*: orienting effect; *E*: executive control effect.

**Table 2:** Attention network scores and relationships between attention networks.

**Effect**	**Value (M±SD)**	***t***_***(43)***_	P*
*A*	−0.091±0.049	−12.346	<0.0001
*O*	0.133±0.064	13.794	<0.0001
*E*	0.125±0.063	13.113	<0.0001
*A→O*	0.650±1.232	3.501	0.0011
*O→A*	−0.545±0.841	−4.300	<0.0001
*A→E*	0.596±1.274	3.104	0.0034
*E→A*	−0.505±0.772	−4.340	<0.0001
*O→E*	1.794±3.005	3.961	0.0003
*E→O*	0.804±0.885	6.027	<0.0001

^*^Two-tailed. M: mean; SD: standard deviation; *X→Y*: the influence from *X* to *Y*.

**Table 3:** The results of main effects and inter-network interactions in interaction blocks.

**Block**	**Effect**	***F***_***(1, 43)***_	***P***
*A* × *O*	*A*	82.783	<0.0001
	*O*	323.663	<0.0001
	Interaction	22.496	<0.0001
*A* × *E*	*A*	115.983	<0.0001
	*E*	219.810	<0.0001
	Interaction	26.800	<0.0001
*O* × *E*	*O*	1083.625	<0.0001
	*E*	231.921	<0.0001
	Interaction	47.813	<0.0001
